# Full Radiology Report through Patient Web Portal: A Literature Review

**DOI:** 10.3390/ijerph17103673

**Published:** 2020-05-22

**Authors:** Mohammad Alarifi, Timothy Patrick, Abdulrahman Jabour, Min Wu, Jake Luo

**Affiliations:** 1 College of Health Sciences, University of Wisconsin-Milwaukee, Milwaukee, WI 53211, USA; malarifi@uwm.edu (M.A.); wu@uwm.edu (M.W.); 2 College of Medical Applied Sciences, King Saud University, Riyadh, SA 11451, USA; 3 College of Engineering, University of Wisconsin-Milwaukee, Milwaukee, WI 53211, USA; Tp5@uwm.edu; 4 Health Informatics Department, Faculty of Public Health and Tropical Medicine at Jazan University, Jazan, SA 45142, USA; Ajabour@jazanu.edu.sa

**Keywords:** full radiology, report, radiology report, patient web portal, social media platforms, quality health care

## Abstract

The aim of this study discusses the gap between the patient web portal and providing a full radiology report. A literature review was conducted to examine radiologists, physicians, and patients’ opinions and preferences of providing patients with online access radiology reports. The databases searched were Pubmed and Google Scholar and the initial search included 927 studies. After review, 47 studies were included in the study. We identified several themes, including patients’ understanding of radiology reports and radiological images, as well as the need for decreasing the turnaround time for reports availability. The existing radiology reports written for physicians are not suited for patients. Further studies are needed to guide and inform the design of patient friendly radiology reports. One of the ways that can be used to fill the gap between patients and radiology reports is using social media sites.

## 1. Introduction

The patient portal is an online instrument that provides patients with secure and direct access to their medical records [1,2]. There is a need to improve communication between patients and their doctors [3]. A better communication tool is also needed to help patients understand their treatment procedures that could lead to better outcomes. In addition, improved communication not only can help patients better understand treatment procedures and result in more favorable outcomes, but also can reduce potential medical errors. There are many ways for patients to communicate with doctors that may or may not be time-consuming or costly. One of these methods involves scheduling face-to-face appointments with physicians to discuss issues and receive advice. A patient could also call the clinic or hospital and ask to speak directly to a physician or nurse to have questions answered. These methods can be time-consuming and could impact the quality of treatment. Therefore, new technologies are developed to improve the health care communication and information delivery, such as electronic health records (EHRs), personal health records (PHRs), and health information exchanges (HIE). These systems provide information and mechanisms to facilitate patients to understand their health conditions. The systems also improve communication between the patient and their medical provider(s). Patient portals offer a convenient way for patients to access their PHR online. Some patient portals also provide a rich amount of information imported from medical records, such as clinical notes, laboratory results, radiology images, scheduled appointments, and medical bills [4,5,6,7]. A study of 129,419 patients who had access to a patient portal found that patients were more likely to view their laboratory results (59.8%) than clinical notes (34.4%) and radiology results (51.2%) [2]. A previous study on this topic found that patients prefer to view their medical information using patient portals and that there was a strong need to improve the current portal system [2]. This project focuses on full radiology reports in the patient web portal. The goal of the paper is to identify why information about radiology is limited in the online portal. The paper also provides suggestions to address this issue [2]. By evaluating the gap between patient preferences and current access to full radiology reports, researchers and developers can design and create a better solution to improve the communication of radiology reports to patients. Over the years, medical centers have always struggled to improve quality and operational efficiency and cut down costs. Despite efforts and using a variety of strategies, many problems arise while delivering quality health care.

## 2. Materials and Methods 

The goal of this study was to understand patients’ needs and preferences related to access to their radiology images and reports. Prior to the literature review, we also called 110 hospitals to inquire about existing practice when it comes to providing patients with radiology images and reports. Later, a literature review was conducted to examine patients’ preferences and needs related to radiology reports.

### 2.1. Context and Existing Status

To enhance our understanding of existing practice prior to the literature review, we contacted 110 US hospitals over the phone to inquire if they provide patients with radiology reports, radiological images, or both. Of the hospitals surveyed, 98 responded that they only provided patients with access to the reports, but not the images themselves. Twelve hospitals stated that the information made available to patients depended on the case. For example, in cases involving breast cancer, brain cancer, or renal cell carcinoma (RCC), a hospital could offer all available information to the patient. Some health care providers, such as Mayo Clinical Health, require patients to receive doctor permission before obtaining access to certain medical images, such as an electrocardiogram (ECG). This health care system generally offers full radiology reports.

### 2.2. Search Strategy and Criteria

Google Scholar and PubMed were used to find studies that discuss the gap between the patient web portal and the full radiology report. The keywords used were patient portal OR patient web portal AND radiology report. We searched for journal articles published within the last five years (after 2015). In addition, the reference lists of the relevant results were also checked.

**Figure 1:**
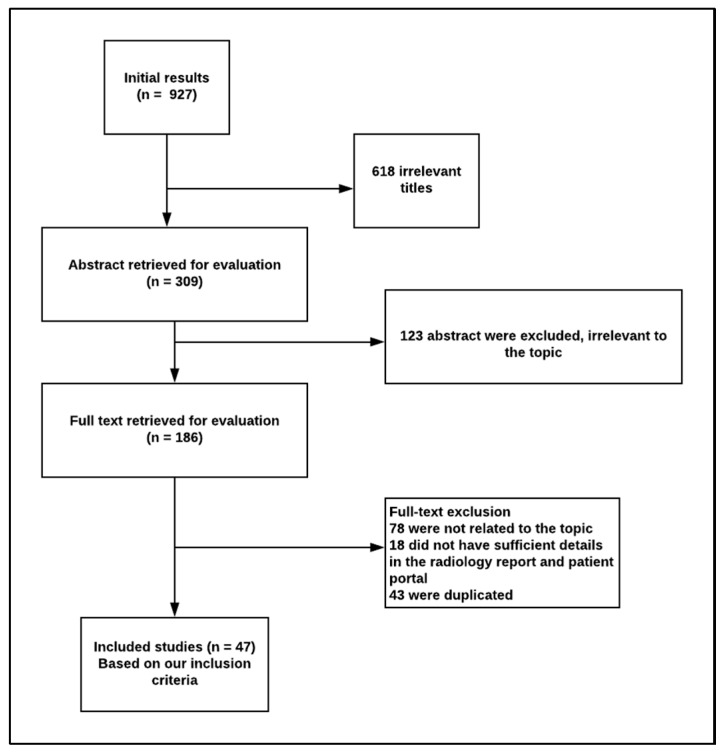
The criteria for inclusion and exclusion.

The initial search resulted in 927 articles that were screened by reviewing the title for its relevance to the topic. After title screening, 618 articles were removed and 307 remained. We reviewed the abstracts of the 309 abstracts and removed 123 for not being relevant to the topic. The full texts of the remaining 184 articles were retrieved for further screening. Of these 184 articles, 47 met our criteria for being relevant to the topic and were further reviewed in detail (Table 1). Our inclusion criteria were: it should be an original study, published within the last five years, relevant to the topic, discuss patients’ needs and preferences of radiology reports, and published in English (Figure 1). 

## 3. Results

### 3.1. Current Radiological Reports and Patient Portal

Dr. David Naeger, the co-director of the Henry I. Goldberg Center for Advanced Imaging Education at the University of California, stated that at this time, most patients prefer to have full access to their medical information and a full radiology report [38]. In the same survey, two-thirds of the participants stated that they preferred having a copy of their full radiology reports and that they wanted to meet with or engage in some form of communication with their physicians to discuss the reports [38]. The author noted that half the participants did not know that radiologists are doctors, but 79% of patients understood that radiologists were specialists at explaining certain medical images [38]. Another study found that 86,400 patients per month out of 234,679 total patients accessed their portal online [18]. This statistic showed that there was an increase in awareness of the portal among the patient population. The current output of the system showed that radiology reports are not generally available through the web portal [38]. Information about radiology reports is generally limited when viewed through the portals [2,9,17,22,39,42,47]. The information currently provided is not easily understood by patients, which is an issue for many radiologists and referencing physicians (RPs). These medical professionals are concerned that patients who can access these reports may experience greater anxiety and engage in a time-consuming follow-up process with their RPs to answer their questions and obtain a greater understanding of the report content [13,28,29,30,38,41,44,52]. To evaluate these issues, many points must be taken into account. 

### 3.2. Social Media and Patient Understanding

The patient questions and comments in social media have been used to understand the patient’s concerns and needs in many fields. As an example, a study conducted to know cancer patients’ needs and preferences for accessing different formats of platforms. They found that 22% of patients wanted a wide range of platforms that were easier to understand, and 25% wanted the platforms to be more accurate [53]. Unfortunately, there is no study at this moment that leverages social media to understand unmet patients’ needs related to reading and interpreting radiology reports. There are many platforms that can be used to collect patients’ questions such as Yahoo!Answers, WebMD community, PatientsLikeMe, Quora and Tumblr.

## 4. Discussion

### 4.1. Benefits of a Patient Portal for Patient Engagement 

Patient engagement is a process that puts a patient at the center of his or her health care [36,47,54]. Patients can be informed about their health conditions in a variety of ways, including receiving calls and messages from doctors, seeing doctors during appointments, or by accessing a patient web portal. Studies have found that many patients who used the health portal at some point in the previous year had a more positive feeling about their health care experiences [37,50]. By accessing personal health records (PHRs), patients can see their health records and will be able to gain a better understanding of their health [37]. When patients have an electronic copy of their medical records, they can share these records with other health care providers to acquire second opinions and advice. A PHR will lead to an increase in the interaction between patients and their health care providers and allow patients to play an important role as a member of their health care team [55]. In a survey, patients with greater engagement with their medical portal reported fewer errors and received higher quality treatment [56].

Patient portals also lead to decreased health care costs by reducing medical errors and improving the patient’s cooperation [56]. An increased understanding of the treatment process could lead to patients making fewer mistakes in their care that could lead to a repeat condition or illness and require the same type of medical treatment [8,11,14,16,19,20,57]. Through increased patient engagement, a health care provider can save time and money. These resources could be spent on the treatment of patients for other issues [56]. Patient engagement is one of the critical requirements for meaningful use. Health information and patient engagement exchange are required in stage 2 of meaningful use [2]. The most significant factor in enhancing communication between physicians and patients involves supporting patient engagement with tools such as a patient web portal. These tools can deliver specific and generic medical information to patient users [36]. For example, when a patient sees images and reads the interpretations for the images of an indication, such as a lung cancer tumor, the patient can then gain a better understanding of what the cancer condition looks like. This information of imaging reports will form a foundation of information that a health care provider can build on in future visits. The goal of achieving full patient engagement using medical record access is important but remains something that is far from becoming a universal reality [54].

### 4.2. Impacts of the Health Communications Objectives

Health communication objectives have varied benefits within the process of giving health care services. Some of the benefits of the scientific providing contribution towards developing a shared decision making process between the providers and patients include the following [9,18,21,28,31]. The objective contributes to improving the quality of health care as well as health care safety. During shared decision making, clinicians and patients work in unison in coming up with agreements regarding the care plans, tests, and treatments to be employed that are based on clinical evidence [58]. This factor makes it possible to balance or reduce risks and then anticipated health outcomes, which are by the patient’s values and preferences [38,59]. When the health outcome expectations of the patients are met, health care safety and quality are believed to have been prevalent [60].

The benefits of developing social support networks include enhancing care at home and within the community. The majority of the moves that are aimed at improving social support are mostly directed at the mothers, as they are seen as a group at risk, which starts with carrying the burden of pregnancy. Elements used in building social support networks include health information and health education which tend to give more attention to enhancing parenting skills, which makes it possible to attain better health outcomes for children [43,61].

The objective of providing accessible, actionable and accurate health information that is patient tailored and targeted is essential as it raises the efficiency of public health service and health care delivery [57]. Health information resources are critical to producing health information that helps improve the efficiency of public health services and health delivery, as it enhances care outcomes for families and individuals. Consumer health informatics have been able to assist practitioners with resources that aid towards achieving tailored health care services, which improves efficiency within the public health service [47]. As the public is increasingly getting involved in health care, consumer health informatics are playing a vital role in linking up the digital divide as well as backing up the ability of the consumers to author and understand health information [6].

A proper understanding of tailored health information enables caregivers to know what they need to provide quality health care services. Tailored data improve the quality of care delivery, as patient satisfaction is likely to be high [62]. In the event that there is knowledge of the health care information, all the necessary resources can be gathered. This aspect helps create an environment where there is efficiency in care delivery as all the resources regarding a particular health issue have been collected [63].

### 4.3. Addressing the Objectives

The objectives selected include contributing to developing a shared decision making process between the providers and patients, improving social support networks, and providing accessible, actionable and accurate health information, which is tailored and targeted. On the objective relating to shared decision making, the critical consideration would be to create a platform within the health care environment [64]; a situation where both the patient and the caregivers can come together and be able to analyze different aspect that related to treatment and tests among other health care practices that, at the end of the day, ensure that there are quality health care outcomes. This approach can be implemented within a care setting through coming up with advance care planning talks, decision support counseling, and ensuring patient decision aids. When this strategy is in place, the objective of attaining share decision between caregivers as well as patients [64].

The objective of developing social support networks can be attained by coming up with opportunities that enhance social connectedness, which targets improving health care outcomes [65]. Some of the initiatives that can be used to promote this objective include coming up with education sessions, which tend to provide more knowledge on issues like parental skills to enhance the health of children. These meetings can be held in a health care facility or within community centers to help the communities at risk of disadvantaged social support to achieve quality health.

On the objective of providing accessible, actionable and accurate health information, which is tailored and targeted, the primary focus is on the deliverance of reliable health information that can be used to achieve quality care [57]. For health information to be honest, it has to be accurate, actionable, and accessible. This objective can be achieved through ensuring that there are reliable health information resources such as tools and standards like routine community and facility reporting systems, health statistics and data like the Global Health Observatory (GHO), and national evaluation and monitoring guidance, like in [66].

### 4.4. Quality Initiatives and Patient Satisfaction

One incentive geared towards quality is the use of computerized information technology [67]. Together with electronic health care records (EHRs), this system will make it easier for health care providers to assess correct data and in turn, patients will receive better treatment [68]. The EHRs will help to reduce or prevent any medical errors while improving patient care while inducing an illness diagnosis to a patient [35,46,49,69]. In addition, the EHRs can determine potential health issues and can aid the medical center to prevent any occurrence of entering a wrong diagnosis, hence, creating better results for the patients [70].

Secondly, taking measures that reduce medical errors, readmission, and implementation of ways to minimize errors is a quality initiative for patient satisfaction [71]. The programs that address care practices to reduce readmission and good quality health care implementation have registered health care improvement and have shown improved patients’ outcome. A misunderstanding or poor patient care given on the first visits may be a reason for readmission [72]. These readmissions cause high annual costs [73]. Reduction in readmissions can save costs concerning the patient and the hospitals, which can result in fewer errors, hence improving the patient outcome [73]. At this condition, the patient’s education is of necessity so that patients can follow up on their appointments to avoid readmission [70].

Improving communication by using applications, such as mYhealth in mobile technology, can be another step. There exists a difficulty in communication between the providers and the patients. Current metrics show that hospitals waste billions of dollars because they lack proper communication with providers [73]. Medical centers are evaluating how going mobile can help improve communication. With applications in mobile technology being a priority, it will serve to improve patient safety and increase clinician efficiency [74]. These new ways of technology and treatment will improve the quality of patient outcomes.

### 4.5. Patient Portal Platforms

There are many examples of patient portals on the web, such as Intelichart, the Kaiser patient portal, MyChart, and others [54]. Some of the portals are used by many hospitals, such as MyChart, which covers 99 hospital systems. The need for this type of system is high because patients need tools that can remind them to follow up with their medication schedule, to add personal notes about doctor instructions, and to engage in a convenient dialogue with health providers for issues that may be of concern. Some example applications that address these needs include Dosecast, Mango Health, MedCoach, MediPrompt, MediSafe, MedMory, MyMedSchedule, MyMeds, Pillboxie, PillMonitor, and RxmindMe. They provide different degrees of services to the patients [54]. One application, RxmindMe, allows users to create nine types of reminders including hourly, daily, weekly, and monthly reminders. The application also allows users to download their prescription history and send it via email. Users can download these applications onto their smartphones or tablets to track their health record. This portal allows patients to improve their health by ensuring that the patients correctly follow clinicians’ medication instructions. Patients can also input additional signs that their health is negatively changing including new injuries, weight gain, or skin conditions that occur with no apparent cause. When the portal is updated, the patients can share the portal contents with external parties to obtain a more accurate treatment. 

### 4.6. Limitations in the Patients’ Portal

Patient web portals generally need a lot of work to improve the limitations of these platforms [52]. This paper focuses on access to full radiology reports in web portals and, therefore, will concentrate on the limitations of the portal regarding this issue. Many studies mention that there are issues and limitations in the radiology section of patient web portals [2,24,52]. There are many physicians, radiologists, and patients who are dissatisfied with the radiology portion of the web portal [52]. There are also limitations in the ability to access all radiology reports in the online portal [2]. In addition, the information available is not understandable for many patients because of the complex medical terminology used in the reports [26,52]. A survey of 617 patients was conducted to investigate how many of these patients would prefer to have full access to their radiology reports using the patient portal [38]. The survey found that 65% of patients preferred to have access to their medical images and the radiology interpretation [38]. This contrast between patient performance and the available information in patient portals reveals that there is a gap between them.

### 4.7. Observed Issues

**Figure 2:**
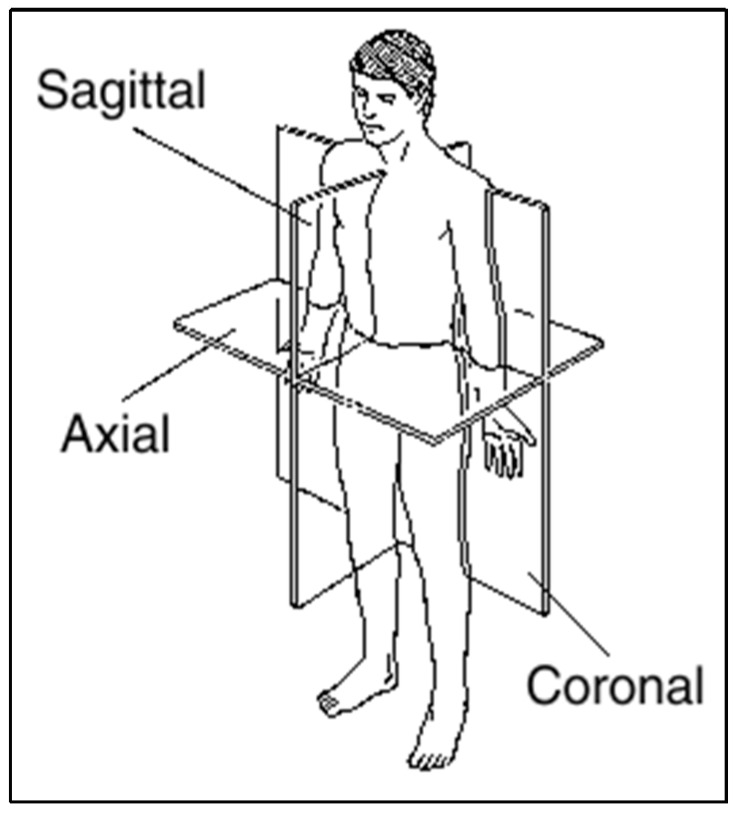
3D Axis set of the human body.

**Figure 3:**
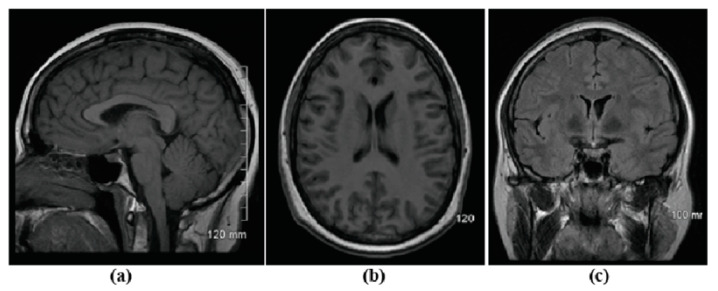
Brain MRI obtained from (**a**) Sagittal Plane, (**b**) Axial plane, and (**c**) Coronal plane.

The information provided by the radiologist department is currently not understandable by all patients and is an issue which concerns referencing physicians (RPs) [52]. The RPs are concerned that patients could misunderstand the images and that these misunderstandings could cause unnecessary anxiety or worry [52]. Radiological images are not easy to understand for those without proper training. Most radiology modalities provide images in white, black, and gray [75]. These colors make the anatomy present in the images difficult to identify and the lack of contrast between the organs in the images is not high. As a result, there could be confusion when differentiating between organs. The reader must also know what to look for when viewing the image, such as knowing that blood would appear as white in the image. If an image of the brain shows a white spot within the brain tissue, this would be an indication of hemorrhage or tumor. The location of the camera concerning the body also provides issues when viewing the radiology images. The sections of the image could be sagittal, coronal, and axial (as shown in Figure 2) [76]. When patients look at the image without the help of a medical professional, they could be confused because of the various views of the same area of the body (Figure 3) [77]. The third barrier that patients may face is that they could not understand the image due to the image’s brightness. Many variables impact the brightness of the image, including contrast, modality type, and electronic window. For example, a patient who has a semi fracture in the foot would have an image with very low brightness to allow the fracture to be seen. Patients could feel that the image is useless or unimportant because the image is very dark. These examples show challenges that decrease the readability of radiology reports and these barriers should be considered and improved as part of the overall improvement in patient access to radiology reports in the patient portal [15].

Most people are not familiar with medical terminology. Radiologists have suggested being given more time to work on the report before uploading the reports to the portal in a form that is more readable and understandable for patients [52]. However, radiologists are concerned about the turnaround time (TAT) for reports [78]. The TAT refers to the time that it takes for radiologists to receive images from PACS, write their interpretation of the images, and then, upload these interpretations into the PACS system. The speed and productivity of radiologists vary based on the individual and the level of the individual. A locum radiologist is a physician who temporarily works in the radiology department while a resident radiologist is a physician who has a contract with the hospital for some time. In the USA, a radiologist must complete 5 years as a resident and a 1- to 2-year fellowship to become a consultant. A consultant radiologist is a physician who can provide final approval for a radiology report written by the locum and resident radiologist. Based on this system, radiology departments have delays in the time it takes to finish all reports [79]. For these reasons, radiology departments do not have additional time or flexibility to work to make the radiology reports easier and more readable for patients [27,32,79]. Some health care providers have a radiology staff shortage that could impact the workflow and TAT. A computerized physician order entry (CPOE) may help to write readable radiology reports for patients in a short amount of time.

## 5. Conclusions

This study shows that there is a gap between information provided in the radiology section of the patient web portal and other sections of the portal. After reviewing the opinions of radiologists, RPs, and patients, we have found that the radiology section needs improvement. The primary concern for radiologists is the time needed to write a simplified report. Before providing images to patients, physicians would like to be able to provide a readable draft to help patients understand the report. The critical factor in making these improvements is to find a way to decrease the TAT by creating ways for radiologists to increase their report writing output while decreasing the time. In addition, it is necessary to investigate the patient’s needs and concerns regarding the radiology report, and questions in social media could be beneficial. These changes would allow patients to receive an understandable radiology report. Additionally, they encourage health providers to provide a radiology section in the patient portal with the same general amount of information as other parts of the portal. 

## Figures and Tables

**Table 1:** Detailed references of the patient portals and the radiology report in the reviewed studies.

Citation	Goal / Purpose	Based on Specific Patient Portal y/n	Study Design	Number of Participants	Types of Participants (Patients / Doctors/ Others)	Finding
Cook TS, et al. 2017 [8]	Impacts the annotations in the radiology reports that included patient-oriented definitions, anatomic illustrations, and hyperlinks to improve patient understanding.	No	Survey	185	Patients	Increased the understanding.
Miles RC, et al. 2016 [2]	To evaluate the frequency with which patients viewed their online radiology reports in relation to in a clinic or laboratory.	Yes	Survey	129,419	Patients	More than half of patients with access to online radiology reports viewed them, with higher viewing rates associated with viewing other types of reports.
Garry K, et al. 2020 [9]	Comparative study of patient satisfaction and understanding of radiology results when received through an electronic patient portal versus direct communication from providers.	No	Survey	1005	Patients	Patients’ understanding of their radiology reports were more through direct provider communication than those who first received their results through the patient portal (26.7% versus 47.8%; *p* < 0.001).
Cho JK, et al. 2020 [10]	To explore patient understanding of the radiology report by using five radiology reporting templates and radiology colloquialisms.	No	Survey	1369	Patients	Adding patient summaries in the report can help increase their comprehension of radiology reports.
Mervak BM, et al. 2016 [11]	To understand patient preferences in the radiology reports by analysis of patient-initiated messages submitted through a web-based electronic patient portal.	Yes	Survey	1489	Patients	Analysis of patient-initiated messages submitted through a patient portal helped to understand the patients’ concerns.
Broman KK, et al. 2015 [12]	To evaluate surgeon and patient acceptance of online postoperative care after general surgical operations.	yes	Survey	50	Patients, Doctors	In general, online postoperative visits were accepted by surgeons and patients.
Rosenkrantz AB, et al. 2017 [13]	Comparing the radiologists, referring physician, and patient interpretations of radiology reports to describe findings of likely low clinical significance.	Yes	Survey	123	Patients, Doctors	Ambiguity in radiologists’ language for incidental low-risk findings may contribute to increased patient anxiety and follow-up testing, warranting greater radiologist attention, and potentially new practice or reporting strategies.
Gunn AJ, et al. 2017 [14]	Providing actual radiology reports to the patients to evaluate their understanding	Yes	Survey	104	Patients	Medical terminologies and longer reports tend to be less well understood.
Martin-Carreras T, et al. 2019 [15]	This study looks to assess the readability of radiology reports.	Yes	Data analysis	108,228	Reports	Only 4% of all reports were readable at the 8th-grade level, which is the reading level of the average US adult.
Vitzthum von Eckstaedt, et al. 2020 [16]	Using the feedback of the patient advisory groups to design a new radiology report for lung cancer.	No	Survey	n/a	Patients	The new report has the potential to serve as a bridge between radiologists and patients, allowing for better patient understanding.
Henshaw D, et al. 2015 [1]	The feasibility of releasing reports to patients before the doctor’s appointment.	No	Survey	508	Patients	Releasing reports to patients was useful before the doctor’s appointment.
Oh SC, et al. 2016 [17]	Will the Prototype System for Patient-Oriented Radiology Reporting (PORTER) improve patients’ understanding of and satisfaction with radiology reports?	No	Survey	300	Reports	PORTER improves patients’ understanding of and satisfaction with radiology reports.
Reicher JJ, et al. 2016 [18]	The impact of the usage of Meaningful Use-compliant electronic heath record (EHR) technology and direct messaging in radiology practice.	Yes	Data analysis	752,496	Messages	It improved radiologist–patient communication.
Short RG, et al. 2017 [19]	Comparing the results of using online crowdsourcing to assess the effectiveness of a Web-Based Interactive radiology report.	No	Survey	193	Patients	Report understanding scores were significantly higher for the interactive web-based than the standard report group (*p* < 0.05)
Martin-Carreras T, et al. 2018 [20]	Comparing MedlinePlus, RadLex, and the PORTER (Patient-Oriented Radiology Reporter) lay-language radiology glossary for the readability of their definitions and coverage of radiology reports.	Yes	Data analysis	10,000	Reports	The readability in PORTER’s glossary definitions was higher than the others.
Qenam B, et al. 2017 [21]	Text Simplification by using Consumer Health Vocabulary can help to increase the readability of the radiology report.	No	Data analysis	792	Reports	The CHV covered a high number of concepts found in the reports but unmapped concepts are associated with locations that are commonly found in radiology reporting
McNamara M, et al. 2015 [7]	To know if the patients prefer to have access to their radiology images or notes.	Yes	Survey	41	Patients	The study found that patients prefer to have access to both their radiology images and notes.
Sadigh G, et al. 2015 [22]	Doctor’s opinion regarding Traditional Text-Only Versus Multimedia-Enhanced Radiology Reporting.	Yes	Survey	402	Doctors	Doctors were satisfied with the format of their current text-only radiology reports and believed that MERR would represent an improvement.
Dy GW, et al. 2018 [23]	To evaluate a patient-centered radiology report (PCRR) for renal ultrasounds in children with hydronephrosis.	Yes	Survey	44	Patients	The patients showed high confidence in the PCRR.
Lye CT, et al. 2019 [24]	To evaluate U.S. hospital compliance with government guidelines and patient straightforward entry to imaging studies.	No	Survey	81	Hospitals	All 80 hospitals provided imaging studies on CDs. Only 8% of hospitals by email and three (4%) via an online patient portal.
Pahade JK, et al. 2018 [25]	To know what information patients or caregivers found useful before an imaging examination.	No	Survey	1542	Patients	Delivery of pre-examination information for imaging examinations is suboptimal, with half of the patients and caregivers seeking information on their own.
Short RG, et al. 2018 [26]	To determine the readability of language used in chest Computer Tomography reports to explain a “normal” thyroid gland.	Yes	Data analysis	11,357	Chest CT (reports)	The language used by radiologists to explain a normal thyroid gland in chest Computer Tomography reports is complex and variable.
Yi PH, et al. 2019 [27]	To evaluate the readability of the lumbar spine in the MRI reports.	Yes	Data analysis	110	Lumbar spine (reports)	The study found that the lumbar spine in the MRI reports are written at a level too high for the average person to comprehend.
Kemp JL, et al. 2017 [28]	The opinion of radiologists regarding direct communication with their patients.	No	Survey	694	Doctors	89% agreed that they should have direct communication with their patients.
Alpert JM, et al. 2018 [29]	To evaluate the current content of oncology in the patient portal.	No	Semi-structured interviews	60	Patients, Doctors	Most of the participants were relatively comfortable with this manner of disclosure but still preferred direct communication.
Mityul MI, et al. 2018 [30]	To know how patients and radiologists understand the commonly used phrases within the radiology report.	No	Survey	113	Patients, Doctors	There is a huge difference between patients and doctors in terms of understanding the medical terms in the radiologic report.
Choudhry A, et al. 2015 [31]	To evaluate the current content of the Biopsy Result in the patient portal.	Yes	Survey	301	Patients	Most of the patients preferred to have direct communication with their doctors by telephone.
Brook OR, et al. 2015 [32]	To compare structured radiology reports versus nanostructured reporting and the effects of both reports on subjective assessment of resectability.	No	Survey	120	Reports	Surgeons were more confident in regards to the structured radiology reports.
Hoang JK, et al. 2018 [33]	The affective of applying American College of Radiology Thyroid Imaging Reporting and Data System (ACR TI-RADS) criteria in the number of thyroid nodules recommended for biopsy.	Yes	Survey	100	Thyroid nodules (reports)	ACR TI-RADS criteria decreased the number of thyroid nodules recommended for biopsy.
Balthazar P, et al. 2017 [34]	To study the impact of trainee involvement and any factors on addendum rates in radiologic reports.	Yes	Data analysis	129,033	Reports	Trainees helped to decrease the addendum rates in radiology report.
Rosenkrantz AB, et al. 2016 [35]	To evaluate information about radiology practices on public transparency Web sites.	Yes	Data analysis	8	Web sites	Transparency Web sites had a lesser extent of service quality and information.
Patmon FL, et al. 2016 [36]	To evaluate using interactive patient engagement technologies (iPET) by nurses.	Yes	Survey	38	Nurses	Nurses who received sufficient training on the iPET system were more comfortable with iPET.
Giardina TD, et al. 2015 [37]	Opinions of patients who have chronic diseases regarding their results in the patient portal.	Yes	Interview	13	Patients	They have several concerns that affected their experience.
Cabarrus M, et al. 2015 [38]	To know patients’ preferences for receiving their radiologic report results.	Yes	Survey	617	Patients	64% of patients want to have copy of their results or online access.
Fang J, et al. 2018 [39]	To know doctor’s experiences with patient interactions in the era of open access of patients to imaging reports.	Yes	Survey	128	Staff and trainee doctors	Most of the respondents found interactions with patients to be a satisfying experience.
Sorondo B, et al. 2016 [40]	To evaluate the patient self-reported screening tool in a patient portal and user experience in primary care.	Yes	Survey	72	Patients	Patients can effectively use their portals to complete the patient report.
Laccetti AL, et al. 2016 [41]	To examine patterns of use of patient portals by clinic employees at a National Cancer Institute-designated comprehensive cancer center.	Yes	Data analysis	289	Nurses, Ancillary staff, Clerical/managerial staff, Doctors, Advanced practice providers	All the staff efforts that related to a patient portal has improved markedly over time, especially among nursing staff.
Woollen J, et al. 2016 [42]	To investigate patient experience with browsing their laboratory test results and radiology reports on a tablet or computer from the patient portal.	Yes	Semi-structured interviews	14	Patients	Providing a tablet computer may enhance satisfaction, lower anxiety, and increase understanding of their health conditions.
Edwards EA, et al. 2019 [43]	To know parent preferences for pediatric radiology patients.	Yes	Survey	n/a	Parents	The majority of parents prefer to receive the radiology report from a radiologist in-person.
Jung HY, et al. 2017 [44]	This article examined differences in access to text-only reports compared with radiology images through a health information exchange system by health care professionals.	Yes	Data analysis	1670	Doctors and non-doctors	Radiologists, orthopedists, pulmonary disease specialists, and surgeons accessed imaging more often than text-based reports only.
Zide M, et al. 2016 [45]	The effect of patient health literacy in radiology on perceived portal usability.	No	Survey	500	Patients	Those who have more medical conditions have a greater preference for patient portals.
Johnson EJ, et al. 2017 [46]	To evaluate the content of the private practice radiology facilities in the USA.	Yes	Survey	50	Private practice radiology facility	The quality of the content was low.
Kelly MM, et al. 2017 [47]	To know the parent’s opinion of using the inpatient portal application on a tablet computer that presents information about a child’s hospital stay.	Yes	Survey	296	Parents	In general, parents were satisfied with the inpatient portal.
Wildenbos GA, et al. 2018 [48]	To know the opinion of the older adult patients using a patient portal.	No	Survey	10,679	Older adult patients	The majority indicated that they prefer to review their medical information and appointments by the portal.
Alper DP, et al. 2020 [49]	To assess the impact of a reports template quality improvement (QI) initiative on the use of preferred phrases for connecting normal findings in structured abdominal CT and MRI reports.	Yes	Data analysis	44,680	Radiology reports	A QI intervention decreased the use of equivocal terms and increased the use of preferred phrases when connecting normal findings in abdominal MRI and CT reports.
Mishra VK, et al. 2019 [50]	To analyze the patient’s perceptions after being given access to specialist’s notes and primary care via the patient portal.	No	Survey	6439	Patients	The study confirms that the patients who have access to their specialists’ online medical records and primary care perceived benefits of OpenNotes.
O’Leary KJ, et al. 2016 [51]	To evaluate health care provider and patient perceptions of a patient portal and identify opportunities to enhance the current design.	Yes	Semi-structured interviews	18	Patients	Optimizing the patient portal will require attention to the format of information provided, including type and timing.
